# Editorial: Plant Transformation

**DOI:** 10.3389/fpls.2022.876671

**Published:** 2022-04-18

**Authors:** Horacio Esteban Hopp, German Spangenberg, Luis Herrera-Estrella

**Affiliations:** ^1^Departamento de Fisiología, Biología Molecular y Celular, Facultad de Ciencias Exactas y Naturales, Universidad de Buenos Aires (UBA), Buenos Aires, Argentina; ^2^Instituto de Agrobiotecnología y Biología Molecular (IABIMO), UEDD INTA-CONICET, Hurlingham, Argentina; ^3^AgriBio, Agriculture Victoria, La Trobe University, Melbourne, VIC, Australia; ^4^Plant and Soil Science Department, Institute of Genomics for Crop Abiotic Stress Tolerance, Texas Tech University, Lubbock, TX, United States; ^5^Unidad de Genómica Avanzada/LANGEBIO, Centro de Investigación y de Estudios Avanzados, Irapuato, Mexico

**Keywords:** plant transformation, gene editing, selectable markers, transient expression, transformation protocol

Plant transformation provides a key tool for much basic research, such as the study of gene functions and interactions, protein–protein interactions, developmental processes, as well as applications for crop improvement and the development of plant bioreactors to produce vaccines. Efficient and reproducible transformation technologies are not only essential for the development of transgenic plants but also critical for other applications like transient gene expression studies and gene editing.

*Agrobacterium tumefaciens* was first identified in 1907 as the etiological agent of crown gall disease. The bacterial factor responsible for tumor induction was described in the ‘70s: a DNA plasmid called the Ti plasmid by Zaenen et al. (1974). Transposon mutagenesis was used to dissect the functional regions of the plasmid Ti, and two main regions were identified: (1) a segment of the Ti plasmid, denominated T-DNA, which is transferred into plant cells and integrated into the plant genome, and (2) a virulence region that provides all functions necessary for T-DNA transfer (for a review see Gelvin, 2000). The Ti plasmid was engineered by removing the genes responsible for tumor induction and replaced by dominant selectable markers to produce transgenic plants (Herrera-Estrella et al., 1983; Zambryski et al., 1983; De Block et al., 1984). Two repetitive 25 bp sequences at the right and left border were reported as essential for the transfer of the T-DNA (Wang et al., 1984). During the complex process of T-DNA integration into the plant genome, sometimes border T-DNA sequences are not recognized as limiting and vectors get integrated as well, particularly in the case of the left border. To reduce the integration frequency of undesired backbone vector DNA segments Sahab and Taylor incorporated multiple left border repeats. Molecular analyses confirmed a 2-fold reduction of vector sequence integration when triple left borders in three different transformation systems were tested including cassava transformation.

Even though the first transgenic plants were produced in the early ‘80s, not all plant species are transformed as easily as model species, particularly when it comes to some economically important crop species. Some plants are still considered as difficult or recalcitrant to transformation. Almost every plant species has a specific transformation protocol that slowly evolved over the years and has not been updated in the last two decades except for the methodology section of published papers. Protocols were modified to facilitate new breeding techniques like gene editing, and some of the latest methodological improvements include breakthrough advancements like the use of developmental regulator genes and tissue-culture independent gene editing protocols. This Research Topic provides a collection of reviews and original research articles on the recent advancement of plant transformation and gene editing of different crops. Below we briefly describe the original papers are reviews that integrate this Research Topic. Maize, perhaps the crop species with most transgenic commercial traits incorporated so far and for which many new approaches to improve its engineering, including genome editing, is critically summarized by Carvalho-Teixeira-Yassitepe et al.. In contrast, the genetic transformation of Teosinte the most closely related wild species of maize (phylogenetically speaking) did not evolve as fast as its domesticated relative. In this context, Zobrist et al. report an innovative protocol by using whorl segments of seedlings germinated from mature seeds of *Zea parviglumis*.

As mentioned above, efficient genetic transformation is currently performed routinely for many plant species, but there are still many recalcitrant species. Even in species where transformation was successfully achieved, some agronomically important genotypes remain reluctant, thus slowing down breeding programs since the obtention of new engineered varieties normally requires the introgression of transgenic or gene-edited traits into elite germplasm. Several reports in this Research Topic deal with this issue by improving tissue culture conditions. For example, pluronic F-68 (PF-68) is a non-ionic surfactant used in plant tissue culture as a growth additive. Kok et al. report that supplementation with specific PF-68 concentrations enhances callus proliferation in recalcitrant rice cultivars. Chinese cabbage is another example of a recalcitrant crop. Sivanandhan et al. report stable *Agrobacterium tumefaciens*-mediated transformation for Chinese cabbage cv. Kenshin by employing antioxidants in the co-cultivation and subsequent regeneration media. The *Asteraceae* family is the largest and most diversified family of the Angiosperms and includes economically important crops such as lettuce and sunflower. There is a sharp contrast between lettuce that has easily adapted to tissue culture and transformation and sunflower, which is much trickier and difficult to transform. The peculiarities of their genetic transformation protocols are described by Darqui et al..

Grapevine has been considered a recalcitrant crop to produce transgenic plants as several other woody perennials. The ability to regenerate plants from transformed explants is considered the main obstacle in the process. State-of-the-art technologies to improve grapevine transformation and regeneration are reviewed by Campos et al.. Switchgrass is a grass of importance for biofuel production. Upland switchgrass cultivars are recalcitrant to genetic transformation. As for the case of the rice cultivar below, Xu et al. report improved transformation methods for two important upland cultivars. Speaking of forage crops and grasses, dallisgrass is a very important apomictic pasture in South America and other temperate warm regions of the world. In the paper authored by Schrauf et al., the transformation methodology status of cultivars of *Paspalum dilatatum* transformation is reviewed, and the authors propose this species as a model for molecular breeding in C4 perennial forage species. Although apomixis (asexual reproduction by seeds) is a pursued trait in grasses because the introduced transgene becomes immediately fixed in a highly adapted genetic background capable of large-scale clonal propagation, it may present a serious constraint for conventional breeding. Another review (Bellido et al.) addresses this paradox by describing the potential molecular pathways involved in apomixis determination and how these pathways could be used for both conventional and molecular breeding.

Many crops belong to polyploid species and require the search of systems to identify new dominant markers. A perspective article from Jozefkowicz et al., proposes the use of the *Tnt1* element as a candidate to identify dominant mutations in allogamous tetraploid cultivated alfalfa. They illustrate this potential by showing that a single allelic mutation in the MsNAC39 gene produces multifoliol leaf plants in alfalfa. Sugarcane is another complex polyploid (and aneuploid) species. Modern sugarcane cultivars have extremely large genomes originating from artificial interspecific crosses making traditional breeding extremely difficult. The review authored by Budeguer et al. summarizes the current techniques and state of the art in sugarcane transformation. Potato is another well-studied polyploid crop of global importance that is not apomictic but clonally propagated like apomictic grasses, sharing the difficulties in conventional breeding. Thus, genetic engineering provides the opportunity to introduce/switch-off genes of interest without altering the allelic combination that characterizes successful commercial cultivars or to induce targeted sequence modifications by genome editing. The review by Nahirñak et al. summarizes the latest developments in the field.

Citruses are among the most prevailing fruit crops produced worldwide. As for the examples mentioned above, conventional methods are difficult because of prolonged juvenile periods, complex reproductive stages, occasional low fertility, self-incompatibility, parthenocarpy, or polyembryony. Genetic engineering technologies offer alternative approaches for overcoming these difficulties. The review by Conti et al. provides a detailed overview of the currently used strategies for the development of genetically modified citruses.

Forest-tree breeding using transgenic technology is still in its early stages compared to annual plants except for a few model species. In the review by Yin et al., the later advances in transgenic technology of forest trees and their application for trait improvement are discussed. Some forest species are widely used in agroforestry plantations for soil stabilization, ecosystem rehabilitation, etc. This is the case of *Casuarina equisetifolia*. In the study authored by Ren et al., more efficient and rapid regeneration systems based on stem segment explants are reported.

Another important aspect of molecular breeding is the identification and characterization of complex industrial traits. This is the case of fiber length, strength, and other fiber quality parameters in cotton. Razzaq et al. present an example of gene editing to improve fiber quality parameters appreciated by the textile industry.

*Cannabis sativa* produces unique phytocannabinoids and there are scarce if any reports of *in vivo* engineering targeting the cannabinoid biosynthesis genes. Matchett-Oates et al. report the successful modulation of cannabinoid biosynthesis genes using RNAi via agroinfiltration, enabling functional genomics of targeted cannabinoid biosynthesis genes.

Finally, another woody plant was successfully transformed with *Bacillus thuringiensis* toxin gene Cry10Aa to induce coffee berry borer (CBB) resistance, as reported by Valencia-Lozano et al.. Bioassays using transgenic fruits challenged with CBB larvae and adults significantly reduced seed damage to less than 9%, providing a powerful CBB control tool for coffee production.

## Author Contributions

HH was a guest associate editor of the Research Topic and wrote the paper text. GS and LH-E were guest associate editors of the Research Topic and edited the text. All authors contributed to the article and approved the submitted version.

## Funding

HH was supported by by the Agencia Nacional de Promoción Científica y Tecnológica, Argentina (ANPCYT: PICT 2018-02926).

## Conflict of Interest

The authors declare that the research was conducted in the absence of any commercial or financial relationships that could be construed as a potential conflict of interest.

## Publisher's Note

All claims expressed in this article are solely those of the authors and do not necessarily represent those of their affiliated organizations, or those of the publisher, the editors and the reviewers. Any product that may be evaluated in this article, or claim that may be made by its manufacturer, is not guaranteed or endorsed by the publisher.
